# (*E*)-2-Hydroxy­naphthalene-1-carb­al­de­hyde semicarbazone

**DOI:** 10.1107/S160053680901174X

**Published:** 2009-04-18

**Authors:** Hua-Jie Xu, Na-Na Du, Xue-Yue Jiang, Liang-Quan Sheng, Yu-Peng Tian

**Affiliations:** a Department of Chemistry, Anhui University, Hefei 230039, People’s Republic of China; b Department of Chemistry, Fuyang Normal College, Fuyang, Anhui 236041, People’s Republic of China

## Abstract

The title compound, C_12_H_11_N_3_O_2_, adopts an *E* or *trans* configuration with respect to the C=N bond. There is an intra­molecular O—H⋯N hydrogen bond involving the hydroxyl H atom and an N atom of the hydrazine group. In the crystal structure, mol­ecules are connected *via* N—H⋯O hydrogen bonds, forming a three-dimensional network.

## Related literature

For the potential pharmacological and anti­tumor properties of hydrazones and Schiff bases, see: Karthikeyan *et al.* (2006 ▶); Khattab (2005 ▶); Kucukguzel *et al.* (2006 ▶). For related structures, see: Okabe *et al.* (1993 ▶); Zhang *et al.* (1999 ▶); Xu *et al.* (2009 ▶).
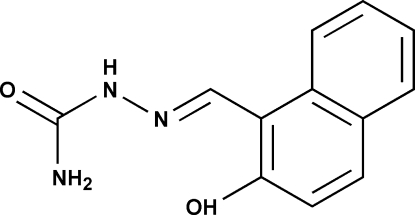


## Experimental

### 

#### Crystal data

C_12_H_11_N_3_O_2_
*M* *_r_* = 229.24
Monoclinic, 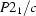

*a* = 16.091 (3) Å
*b* = 4.7350 (9) Å
*c* = 15.776 (3) Å
β = 114.26 (3)°
*V* = 1095.8 (4) Å^3^
*Z* = 4
Mo *K*α radiation
μ = 0.10 mm^−1^
*T* = 283 K
0.20 × 0.10 × 0.10 mm

#### Data collection

Bruker SMART CCD area-detector diffractometer
Absorption correction: multi-scan (*SADABS*; Sheldrick, 1996 ▶) *T*_min_ = 0.988, *T*_max_ = 0.989
6629 measured reflections
2324 independent reflections
1550 reflections with *I* > 2σ(*I*)
*R*_int_ = 0.015

#### Refinement

*R*[*F* ^2^ > 2σ(*F* ^2^)] = 0.037
*wR*(*F* ^2^) = 0.107
*S* = 1.05
2324 reflections
155 parameters
H-atom parameters constrained
Δρ_max_ = 0.19 e Å^−3^
Δρ_min_ = −0.15 e Å^−3^

### 

Data collection: *SMART* (Siemens, 1996 ▶); cell refinement: *SAINT* (Siemens, 1996 ▶); data reduction: *SAINT*; program(s) used to solve structure: *SHELXS97* (Sheldrick, 2008 ▶); program(s) used to refine structure: *SHELXL97* (Sheldrick, 2008 ▶); molecular graphics: *SHELXTL* (Sheldrick, 2008 ▶); software used to prepare material for publication: *SHELXTL*.

## Supplementary Material

Crystal structure: contains datablocks global, I. DOI: 10.1107/S160053680901174X/su2105sup1.cif

Structure factors: contains datablocks I. DOI: 10.1107/S160053680901174X/su2105Isup2.hkl

Additional supplementary materials:  crystallographic information; 3D view; checkCIF report

## Figures and Tables

**Table 1:** Hydrogen-bond geometry (Å, °)

*D*—H⋯*A*	*D*—H	H⋯*A*	*D*⋯*A*	*D*—H⋯*A*
O1—H1⋯N1	0.82	1.83	2.5563 (15)	146
N2—H2⋯O2^i^	0.86	2.01	2.8385 (15)	163
N3—H3*A*⋯O1^ii^	0.86	2.14	2.9871 (15)	171
N3—H3*B*⋯O2^iii^	0.86	2.63	3.0957 (16)	115

Symmetry codes: (i) 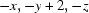; (ii) 
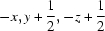
; (iii) 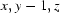.

**Fig. 1.**
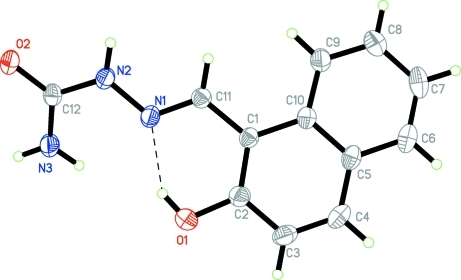
The molecular structure of the title compound, showing 30% probability displacement ellipsoids (H-atoms have been omitted for clarity).

**Fig. 2.**
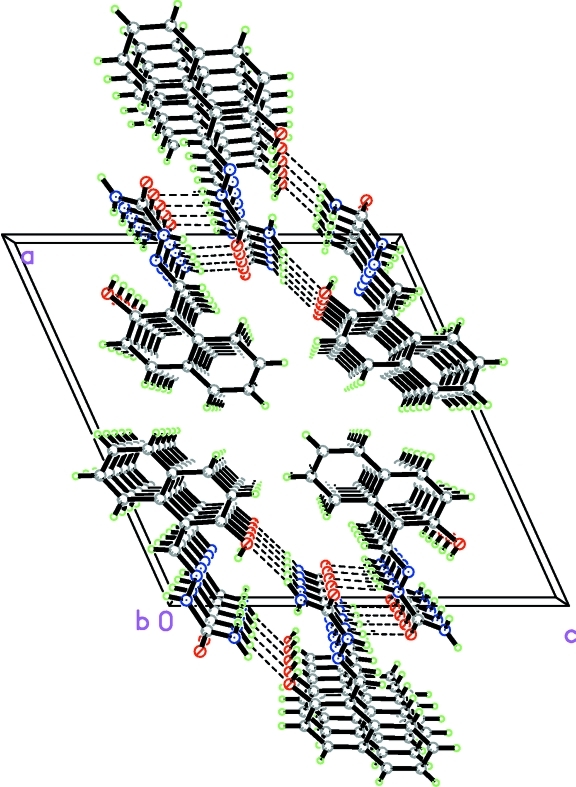
Crystal packing of the title compound viewed along the b axis. The intra- and intermolecular hydrogen bonds are shown as dashed lines (details are given in Table 1).

## supplementary crystallographic information

### Comment

Hydrazones and Schiff bases have attracted much attention for their excellent biological properties, especially for their potential pharmacological and antitumor properties (Kucukguzel *et al.*, 2006; Khattab, 2005; Karthikeyan *et al.*, 2006; Okabe *et al.*, 1993). As we are interested in this field of research (Xu *et al.* 2009), we report herein on the crystal structure of the title compound.

The molecular structure of the title compound is shown in Fig. 1 and geometrical parameters are given in the archived CIF. The molecule has a *trans* configuration about the C=N bond. There is an intramolecular O-H···N hydrogen bond involving the naphthalene hydroxyl sunstituent and atom N1 of the hydrazine group (Table 1).

In the crystal structure symmetry related molecules are connected *via* N-H···O hydrogen bonds so forming a three-dimensional structure (Table 1 and Fig. 2).

### Experimental

The ligand H_2_L was prepared according to the reported procedure (Zhang *et al.*, 1999). A solution of semicarbazide hydrochloride (0.112 g, 1 mmol) in 5 ml of ethanol was added slowly to a solution of 2-hydro-1- naphthaldehyde (0.172 g,1 mmol) in 15 ml absolute ethanol, under heating and stirring. The mixture was refluxed for 3 h, then cooled to rt and left to stand in air for 5 days. Yellow block-shaped crystals were formed on slow evaporation of the solvent.

### Refinement

All the H-atoms were placed in calculated positions [O—H = 0.82 Å, N—H = 0.86 Å, C—H = 0.93 Å] and treated as riding [*U*_iso_(H) = 1.5*U*_eq_(parent O-atom) and = 1.2*U*_eq_(parent C-atom and N-atom)].

### Crystal data

**Table:**

C_12_H_11_N_3_O_2_	*F*(000) = 480
*M**_r_* = 229.24	*D*_x_ = 1.389 Mg m^−^^3^
Monoclinic, *P*2_1_/*c*	Mo *K*α radiation, λ = 0.71073 Å
Hall symbol: -P 2ybc	Cell parameters from 628 reflections
*a* = 16.091 (3) Å	θ = 2.5–15°
*b* = 4.7350 (9) Å	µ = 0.10 mm^−^^1^
*c* = 15.776 (3) Å	*T* = 283 K
β = 114.26 (3)°	Block, yellow
*V* = 1095.8 (4) Å^3^	0.20 × 0.10 × 0.10 mm
*Z* = 4	

### Data collection

**Table:**

Bruker SMART CCD area-detector diffractometer	2324 independent reflections
Radiation source: fine-focus sealed tube	1550 reflections with *I* > 2σ(*I*)
graphite	*R*_int_ = 0.015
φ and ω scans	θ_max_ = 27.0°, θ_min_ = 2.8°
Absorption correction: multi-scan (*SADABS*; Sheldrick, 1996)	*h* = −20→20
*T*_min_ = 0.988, *T*_max_ = 0.989	*k* = −6→4
6629 measured reflections	*l* = −19→19

### Refinement

**Table:**

Refinement on *F*^2^	Primary atom site location: structure-invariant direct methods
Least-squares matrix: full	Secondary atom site location: difference Fourier map
*R*[*F*^2^ > 2σ(*F*^2^)] = 0.037	Hydrogen site location: inferred from neighbouring sites
*wR*(*F*^2^) = 0.107	H-atom parameters constrained
*S* = 1.05	*w* = 1/[σ^2^(*F*_o_^2^) + (0.0607*P*)^2^] where *P* = (*F*_o_^2^ + 2*F*_c_^2^)/3
2324 reflections	(Δ/σ)_max_ < 0.001
155 parameters	Δρ_max_ = 0.19 e Å^−^^3^
0 restraints	Δρ_min_ = −0.15 e Å^−^^3^

### Special details

**Table:**

Geometry. All e.s.d.'s (except the e.s.d. in the dihedral angle between two l.s. planes) are estimated using the full covariance matrix. The cell e.s.d.'s are taken into account individually in the estimation of e.s.d.'s in distances, angles and torsion angles; correlations between e.s.d.'s in cell parameters are only used when they are defined by crystal symmetry. An approximate (isotropic) treatment of cell e.s.d.'s is used for estimating e.s.d.'s involving l.s. planes.
Refinement. Refinement of *F*^2^ against ALL reflections. The weighted *R*-factor *wR* and goodness of fit *S* are based on *F*^2^, conventional *R*-factors *R* are based on *F*, with *F* set to zero for negative *F*^2^. The threshold expression of *F*^2^ > σ(*F*^2^) is used only for calculating *R*-factors(gt) *etc*. and is not relevant to the choice of reflections for refinement. *R*-factors based on *F*^2^ are statistically about twice as large as those based on *F*, and *R*- factors based on ALL data will be even larger.

### Fractional atomic coordinates and isotropic or equivalent isotropic displacement parameters (Å^2^)

**Table:**

	*x*	*y*	*z*	*U*_iso_*/*U*_eq_	
C1	0.23742 (8)	0.2085 (3)	0.15396 (8)	0.0367 (3)	
C2	0.24256 (9)	0.1120 (3)	0.23930 (9)	0.0441 (3)	
C3	0.30716 (10)	−0.0898 (3)	0.29109 (10)	0.0538 (4)	
H3C	0.3094	−0.1497	0.3481	0.065*	
C4	0.36618 (10)	−0.1978 (3)	0.25859 (10)	0.0530 (4)	
H4A	0.4089	−0.3307	0.2939	0.064*	
C5	0.36419 (8)	−0.1126 (3)	0.17185 (9)	0.0435 (3)	
C6	0.42499 (10)	−0.2280 (3)	0.13721 (11)	0.0572 (4)	
H6A	0.4674	−0.3622	0.1722	0.069*	
C7	0.42258 (10)	−0.1462 (4)	0.05361 (12)	0.0623 (4)	
H7A	0.4634	−0.2231	0.0320	0.075*	
C8	0.35881 (11)	0.0531 (3)	0.00052 (11)	0.0571 (4)	
H8A	0.3572	0.1088	−0.0567	0.068*	
C9	0.29852 (9)	0.1678 (3)	0.03161 (9)	0.0477 (4)	
H9A	0.2560	0.2989	−0.0053	0.057*	
C10	0.29938 (8)	0.0915 (3)	0.11853 (9)	0.0377 (3)	
C11	0.17325 (8)	0.4299 (3)	0.10430 (9)	0.0383 (3)	
H11	0.1777	0.5185	0.0537	0.046*	
C12	−0.01922 (9)	0.7854 (3)	0.10281 (9)	0.0405 (3)	
N1	0.11062 (7)	0.5029 (2)	0.13008 (7)	0.0419 (3)	
N2	0.05492 (7)	0.7251 (2)	0.08591 (7)	0.0447 (3)	
H2	0.0671	0.8266	0.0473	0.054*	
N3	−0.04085 (8)	0.6020 (3)	0.15536 (8)	0.0551 (4)	
H3A	−0.0873	0.6333	0.1678	0.066*	
H3B	−0.0083	0.4530	0.1765	0.066*	
O1	0.18694 (7)	0.2086 (2)	0.27791 (7)	0.0616 (3)	
H1	0.1521	0.3272	0.2436	0.092*	
O2	−0.06341 (7)	1.00232 (19)	0.07058 (6)	0.0510 (3)	

### Atomic displacement parameters (Å^2^)

**Table:**

	*U*^11^	*U*^22^	*U*^33^	*U*^12^	*U*^13^	*U*^23^
C1	0.0325 (6)	0.0356 (7)	0.0400 (7)	−0.0004 (5)	0.0127 (6)	0.0012 (6)
C2	0.0426 (7)	0.0456 (8)	0.0445 (7)	0.0005 (6)	0.0183 (6)	0.0036 (6)
C3	0.0547 (9)	0.0555 (9)	0.0458 (8)	0.0047 (7)	0.0151 (7)	0.0146 (7)
C4	0.0425 (8)	0.0467 (9)	0.0575 (9)	0.0073 (7)	0.0080 (7)	0.0094 (7)
C5	0.0317 (7)	0.0378 (8)	0.0543 (8)	−0.0019 (6)	0.0108 (6)	−0.0042 (6)
C6	0.0386 (8)	0.0501 (9)	0.0765 (11)	0.0066 (7)	0.0172 (7)	−0.0063 (8)
C7	0.0479 (8)	0.0625 (11)	0.0841 (12)	0.0004 (8)	0.0347 (8)	−0.0223 (9)
C8	0.0560 (9)	0.0636 (10)	0.0593 (9)	−0.0035 (8)	0.0313 (8)	−0.0097 (8)
C9	0.0436 (8)	0.0519 (9)	0.0478 (8)	0.0035 (6)	0.0191 (7)	−0.0028 (6)
C10	0.0313 (6)	0.0356 (7)	0.0438 (7)	−0.0040 (5)	0.0131 (6)	−0.0052 (6)
C11	0.0371 (7)	0.0381 (8)	0.0389 (7)	−0.0001 (6)	0.0150 (6)	0.0004 (6)
C12	0.0416 (7)	0.0368 (8)	0.0434 (7)	0.0014 (6)	0.0177 (6)	−0.0071 (6)
N1	0.0385 (6)	0.0396 (6)	0.0480 (6)	0.0057 (5)	0.0182 (5)	0.0030 (5)
N2	0.0428 (6)	0.0401 (7)	0.0560 (7)	0.0097 (5)	0.0250 (6)	0.0108 (5)
N3	0.0579 (8)	0.0484 (8)	0.0733 (8)	0.0123 (6)	0.0414 (7)	0.0112 (6)
O1	0.0642 (7)	0.0759 (8)	0.0575 (6)	0.0184 (6)	0.0378 (6)	0.0190 (5)
O2	0.0525 (6)	0.0432 (6)	0.0616 (6)	0.0139 (5)	0.0280 (5)	0.0039 (5)

### Geometric parameters (Å, °)

**Table:**

C1—C2	1.3916 (17)	C8—C9	1.3667 (18)
C1—C10	1.4390 (16)	C8—H8A	0.9300
C1—C11	1.4540 (17)	C9—C10	1.4126 (18)
C2—O1	1.3525 (15)	C9—H9A	0.9300
C2—C3	1.4013 (19)	C11—N1	1.2798 (15)
C3—C4	1.352 (2)	C11—H11	0.9300
C3—H3C	0.9300	C12—O2	1.2336 (14)
C4—C5	1.414 (2)	C12—N3	1.3417 (16)
C4—H4A	0.9300	C12—N2	1.3554 (15)
C5—C6	1.4120 (19)	N1—N2	1.3719 (14)
C5—C10	1.4172 (18)	N2—H2	0.8600
C6—C7	1.360 (2)	N3—H3A	0.8600
C6—H6A	0.9300	N3—H3B	0.8600
C7—C8	1.392 (2)	O1—H1	0.8200
C7—H7A	0.9300		
			
C2—C1—C10	118.31 (12)	C9—C8—H8A	119.7
C2—C1—C11	120.31 (11)	C7—C8—H8A	119.7
C10—C1—C11	121.34 (11)	C8—C9—C10	121.44 (14)
O1—C2—C1	122.52 (12)	C8—C9—H9A	119.3
O1—C2—C3	115.95 (12)	C10—C9—H9A	119.3
C1—C2—C3	121.53 (12)	C9—C10—C5	117.51 (11)
C4—C3—C2	120.36 (13)	C9—C10—C1	123.13 (12)
C4—C3—H3C	119.8	C5—C10—C1	119.36 (11)
C2—C3—H3C	119.8	N1—C11—C1	120.08 (11)
C3—C4—C5	121.27 (13)	N1—C11—H11	120.0
C3—C4—H4A	119.4	C1—C11—H11	120.0
C5—C4—H4A	119.4	O2—C12—N3	122.80 (12)
C6—C5—C4	121.35 (14)	O2—C12—N2	119.96 (12)
C6—C5—C10	119.48 (13)	N3—C12—N2	117.24 (12)
C4—C5—C10	119.17 (12)	C11—N1—N2	118.76 (10)
C7—C6—C5	121.13 (15)	C12—N2—N1	120.39 (10)
C7—C6—H6A	119.4	C12—N2—H2	119.8
C5—C6—H6A	119.4	N1—N2—H2	119.8
C6—C7—C8	119.79 (13)	C12—N3—H3A	120.0
C6—C7—H7A	120.1	C12—N3—H3B	120.0
C8—C7—H7A	120.1	H3A—N3—H3B	120.0
C9—C8—C7	120.64 (14)	C2—O1—H1	109.5
			
C10—C1—C2—O1	−179.71 (12)	C8—C9—C10—C1	179.65 (12)
C11—C1—C2—O1	2.7 (2)	C6—C5—C10—C9	0.63 (18)
C10—C1—C2—C3	1.2 (2)	C4—C5—C10—C9	−179.00 (12)
C11—C1—C2—C3	−176.41 (13)	C6—C5—C10—C1	179.93 (12)
O1—C2—C3—C4	−179.60 (13)	C4—C5—C10—C1	0.30 (18)
C1—C2—C3—C4	−0.4 (2)	C2—C1—C10—C9	178.15 (12)
C2—C3—C4—C5	−0.4 (2)	C11—C1—C10—C9	−4.27 (19)
C3—C4—C5—C6	−179.15 (13)	C2—C1—C10—C5	−1.10 (18)
C3—C4—C5—C10	0.5 (2)	C11—C1—C10—C5	176.48 (11)
C4—C5—C6—C7	179.75 (13)	C2—C1—C11—N1	−12.17 (19)
C10—C5—C6—C7	0.1 (2)	C10—C1—C11—N1	170.30 (11)
C5—C6—C7—C8	−0.5 (2)	C1—C11—N1—N2	175.68 (11)
C6—C7—C8—C9	0.0 (2)	O2—C12—N2—N1	172.35 (11)
C7—C8—C9—C10	0.8 (2)	N3—C12—N2—N1	−7.35 (18)
C8—C9—C10—C5	−1.1 (2)	C11—N1—N2—C12	171.46 (11)

### Hydrogen-bond geometry (Å, °)

**Table:**

*D*—H···*A*	*D*—H	H···*A*	*D*···*A*	*D*—H···*A*
O1—H1···N1	0.82	1.83	2.5563 (15)	146
N2—H2···O2^i^	0.86	2.01	2.8385 (15)	163
N3—H3A···O1^ii^	0.86	2.14	2.9871 (15)	171
N3—H3B···O2^iii^	0.86	2.63	3.0957 (16)	115

Symmetry codes: (i) −*x*, −*y*+2, −*z*; (ii) −*x*, *y*+1/2, −*z*+1/2; (iii) *x*, *y*−1, *z*.

## References

[bb1] Karthikeyan, M. S., Prasad, D. J., Poojary, B., Bhat, K. S., Holla, B. S. & Kumari, N. S. (2006). *Bioorg. Med. Chem.* **14**, 7482–7489. 10.1016/j.bmc.2006.07.015 16879972

[bb2] Khattab, S. N. (2005). *Molecules*, **10**, 1218–1228. 10.3390/10091218 PMC6147684 18007388

[bb3] Kucukguzel, G., Kocatepe, A., De Clercq, E., Sahi, F. & Gulluce, M. (2006). *Eur. J. Med. Chem.* **41**, 353–359. 10.1016/j.ejmech.2005.11.005 16414150

[bb4] Okabe, N., Nakamura, T. & Fukuda, H. (1993). *Acta Cryst.* C**49**, 1678–1680.

[bb5] Sheldrick, G. M. (1996). *SADABS* University of Göttingen, Germany.

[bb6] Sheldrick, G. M. (2008). *Acta Cryst.* A**64**, 112–122. 10.1107/S0108767307043930 18156677

[bb7] Siemens (1996). *SMART* and *SAINT* Siemens Analytical X-ray Instruments Inc., Madison, Wisconsin, USA.

[bb8] Xu, H.-J., Sheng, L.-Q., Liu, Z.-D. & Shao, S.-C. (2009). *Acta Cryst.* E**65**, o666. 10.1107/S1600536809006941 PMC2968967 21582410

[bb9] Zhang, W.-X., Li, J., Si, S.-F., Li, J.-J., Ma, C.-Q. & Jiang, D.-H. (1999). *Chin. J. Inorg. Chem.* **15**, 571–576.

